# Effect of Vitamin B6, B9, and B12 Supplementation on Homocysteine Level and Cardiovascular Outcomes in Stroke Patients: A Meta-Analysis of Randomized Controlled Trials

**DOI:** 10.7759/cureus.14958

**Published:** 2021-05-11

**Authors:** Neetu Kataria, Poonam Yadav, Rajesh Kumar, Niraj Kumar, Mritunjai Singh, Ravi Kant, Vasantha Kalyani

**Affiliations:** 1 Neuroscience, All India Institute of Medical Sciences, Rishikesh, Rishikesh, IND; 2 Obstetrics and Gynaecology, All India Institute of Medical Sciences, Rishikesh, Rishikesh, IND; 3 Nursing, All India Institute of Medical Sciences, Rishikesh, Rishikesh, IND; 4 Neurology, All India Institute of Medical Sciences, Rishikesh, Rishikesh, IND; 5 Internal Medicine, All India Institute of Medical Sciences, Rishikesh, Rishikesh, IND

**Keywords:** cardiovascular disorders, homocysteine, stroke, vitamin b supplementation, vascular death, recurrence, myocardial infarction

## Abstract

Globally, stroke is the fifth-most leading cause of mortality and also the third leading cause of disability. This study aimed to assess the effect of vitamin B_6_, B_9_, and B_12 _supplementation on homocysteine level, risk of stroke, cardiovascular disorders, and vascular death among stroke participants. An extensive literature search was done through PubMed, Medline, Embase, and Clinical key database from 1 January 2000 to 1 January 2020. Effect of vitamin B (B_6_, B_9_, and B_12_) supplementation on homocysteine was assessed with a mean difference in both vitamin and placebo groups. Risk ratio (RR) was calculated for determining the risk of stroke, major cardiovascular disorder, and vascular death by using a fixed-effect model. A total of eight trials with 8513 participants were included for the final analysis. Vitamin B supplementation intervention was found to have a significant benefit in reducing homocysteine in stroke patients (mean difference -3.84; p<0.00001). The intervention of vitamin B supplementation showed a significant risk reduction of 11% for combined risk of stroke, myocardial infarction, and vascular death among stroke patients, 13% for stroke and 17% for vascular death, whereas no beneficial effect was seen for cardiovascular disorders. This meta-analysis demonstrated up-to-date evidence on the beneficial effect of vitamin B supplementations in reducing homocysteine and preventing the combined risk of stroke, myocardial infarction, and vascular death among stroke patients.

## Introduction

Stroke is the third-leading cause of disability among the elderly population. "Stroke can be defined as the sudden death of brain cells due to lack of oxygen or blockage or rupture of an artery to the brain or eye. Globally 70% of strokes were occurring in low-middle income countries" [1]. According to American Heart Association statistics, stroke ranked fifth for causing death in the US, causing 146,383 deaths in 2017. Statistics suggested 6.2 million deaths attributable to cerebrovascular disease worldwide, including 2.7 million deaths from ischemic stroke, 3 million deaths from an intracerebral hemorrhage, and 0.4 million deaths from subarachnoid hemorrhage. In 2016, on average, the rate of death by stroke was one every 3 minutes and 35 seconds. Globally, several countries in Eastern Europe, Africa, and Central Asia have the highest mortality rates due to stroke [2]. Stroke is a leading cause of long-term disability. Stroke reduced mobility of more than half of stroke survivors among the over-65 years age group. In 2018, stroke led to one in every six deaths from cardiovascular disease (CVD) [2]. 

Currently, neurologists focus on addressing modifiable risk factors such as sedentary lifestyle, smoking, and unhealthy dietary patterns that lead to vitamin deficiency and hyperhomocysteinemia. Particularly, three vitamins -- B6, B9, and B 12 -- help in the breakdown of homocysteine amino acid and change it into other essential substances that our body needs. Deficiency of vitamin B12 and folic acid may lead to hyperhomocysteinemia [3]. Several trials have been undertaken on folic acid supplementation to reduce stroke risk by reducing homocysteine levels among cardiovascular disease participants. High levels of homocysteine have already proven an independent factor for causing premature cardiovascular disorders [4]. According to some prospective studies, elevated homocysteine enhances atherosclerosis through increased oxidant stress, impaired endothelial function, and induction of thrombosis, and increased the risk of cardiovascular disease by twofold and the risk of cerebrovascular disease to a lesser degree [5]. Previous Mendelian randomization studies involving homocysteine metabolism concluded that people who were homozygous for the wild-type allele (CC) of the *MTHFR *gene, compared to homozygous for the mutant allele (TT), had 1.93-2.7mol/L [6-7] or 25% higher homocysteine concentrations [6-8], a 26% higher risk of stroke [6-8], and a 16% higher risk of coronary heart disease (CHD) [7]. Homocysteine can build up in the arteries, which may increase the risk of blood clots, heart attack, and stroke. Vitamin B 6, B12, and folic acid are essential for reducing total homocysteine and reverse endothelial dysfunction induced by high total homocysteine levels [8-9]. In a few prospective studies, total homocysteine levels >10.2µmol/L were found to be associated with the doubling of vascular risk, whereas >20µmol/L was associated with an eight to nine-fold increase in vascular risk [10]. Observational studies indicated that for every 5µmol/L rises in homocysteine levels, there is a 32% increased risk of ischemic heart disease and a 59% increased risk of stroke [4].

The researchers noted that the impact of lowering the total homocysteine level on preventing major cardiovascular events with stroke and transient ischemic attack is unknown. The researchers explored variations in interventions, either with only folic acid or combined effect of with vitamin B6, B9, B12. Exclusive effects of combined vitamin B6, B9, and B12 versus placebo were not addressed on homocysteine among stroke patients. Hence, this meta-analysis planned to assess the combined effect of vitamin B6, B9, and B12 on homocysteine levels and its preventive effect on stroke recurrence, cardiovascular disorders, and vascular death among stroke patients.

## Materials and methods

**Figure 1:**
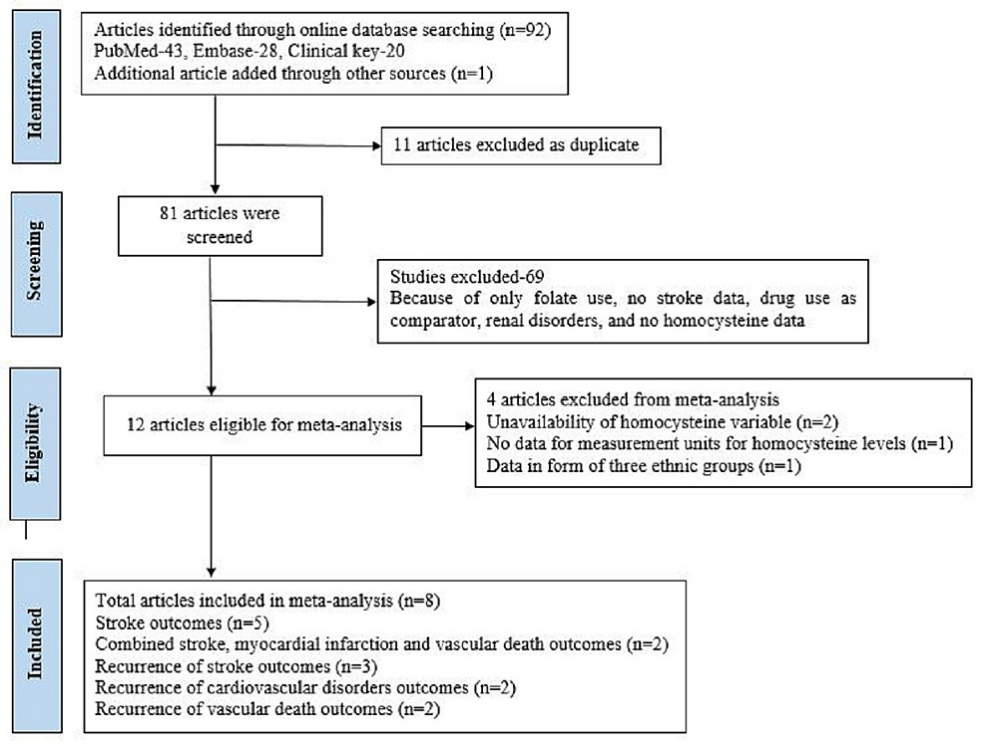
PRISMA PRISMA: Preferred Reporting Items for Systematic Reviews and Meta-Analysis

We followed Preferred Reporting Items for Systematic Reviews and Meta-Analysis (PRISMA) 2020 [11] guidelines to assess the effect of vitamin B6, B9, and B12 on homocysteine levels, risk of recurrence of stroke, major cardiovascular disorders, and vascular death among stroke patients (Figure 1).

Data sources and selection criteria

Online-published trials were searched from PubMed, Medline, Embase, and Clinical Key, published from 1 January 2000 to 1 January 2020 (see Appendices for the search strategies). The language was restricted exclusively to English, and only studies done on human subjects were incorporated. The PICO (population, intervention, control, and outcomes) format was used for the search strategy, with P = stroke, I = vitamin B6, B9 and B12, C = placebo/standard routine therapy, O = homocysteine, recurrence of stroke, major cardiovascular disorders, vascular death. The Medical Subject Headings (MeSH) terms used were population “stroke” along with intervention “vitamin B6”, “vitamin B9”, “vitamin B12”, comparator group “placebo”, with outcome variables “homocysteine”, “recurrence of stroke”, “recurrence of cardiovascular disorders”, and “vascular death”, The lists of references from the included studies were assessed for any additional trials.

Study selection

Two authors (NK and PY) independently screened titles and abstracts and identified published trials. The study design included published randomized controlled trials (RCTs) with a duration from 1 January 2000 to 1 January 2020 with the English language. The study participants affected with stroke (any type); the treatment of combined vitamin B6, B9, and B12 versus placebo; and both the interventional and control groups reported study endpoint in terms of homocysteine level; several events of stroke, cardiovascular disorders, and vascular death separately or combined. The exclusion criteria included studies with stroke patients with any renal disorders and only cardiovascular disorders. The summary of methodological quality of judgments about each methodological item has been displayed as per GRADEpro software (McMaster University and Evidence Prime Inc., Hamilton, Ontario, Canada).

**Table 1:** GRADEpro summary of findings *Estimated risk difference; ^a^Blinding of outcome assessment; ^b^Heterogeneity; ^c^Inconsistency CI: confidence interval; MD: mean difference; MI: myocardial infarction; RR: risk ratio; RCT: randomized controlled trial

Outcomes	Anticipated absolute effects^*^ (95% CI)		Relative effect (95% CI)	№ of participants (studies)	Certainty of the evidence (GRADE)
	Risk with Placebo	Risk with Vitamin			
Homocysteine	The mean homocysteine was 0	MD 3.84 lower (4.17 lower to 3.51 lower)	-	2274 (5 RCTs)	⨁⨁⨁⨁ HIGH
Recurrence of stroke	102 per 1,000	89 per 1,000 (78 to 100)	RR 0.87 (0.77 to 0.98)	9900 (3 RCTs)	⨁⨁◯◯ LOW ^a, b^
Recurrence of cardiovascular diseases	28 per 1,000	28 per 1,000 (22 to 36)	RR 1.02 (0.80 to 1.29)	9627 (2 RCTs)	⨁⨁⨁◯ MODERATE ^c^
Vascular death	99 per 1,000	82 per 1,000 (72 to 93)	RR 0.83 (0.73 to 0.94)	9627 (2 RCTs)	⨁⨁⨁◯ MODERATE ^c^
combined stroke, cardiovascular events-MI, vascular death	173 per 1,000	154 per 1,000 (140 to 168)	RR 0.89 (0.81 to 0.97)	9627 (2 RCTs)	⨁⨁◯◯ LOW ^a, c^

Outcome measures

Both the independent authors (NK and PY) explored primary and secondary outcomes from included trials, including study characteristics and participants' profiles. The primary outcome is to assess the effect of vitamin B6, B9, and B12 on homocysteine levels, and the secondary outcome is to assess the effect of vitamin B6, B9, and B12 supplementation on stroke recurrence, major cardiovascular disorders, and vascular death among stroke patients. 

Data extraction

The two independent authors (NK and PY) performed data extraction from the selected trials. Any discrepancies in eligibility for inclusion were resolved by discussion among the authors. After the selection of each trial, the information extracted was tabulated in the form of the trial name, author name and year of publication, the number of participants, experimental and control groups, mean age, percentage of male participants, treatment for an interventional group and control group, follow-up period, baseline and follow-up levels of homocysteine, events of stroke, and cardiovascular disorders. Any required clarifications were resolved by contacting the author via emails.

Assessment of risk bias and quality assessment

**Figure 2:**
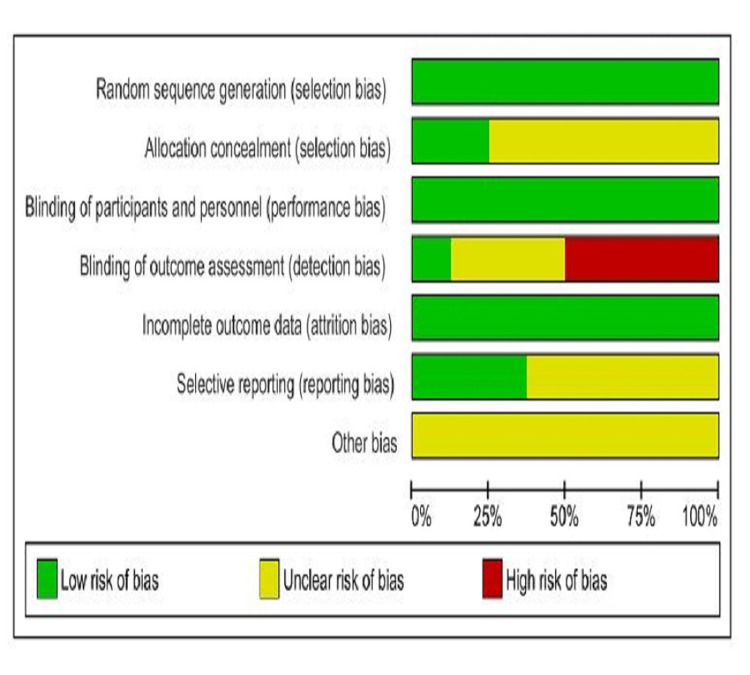
Risk of bias graph Risk of bias graph: review author's judgements about each risk of bias item for each included study.

**Figure 3:**
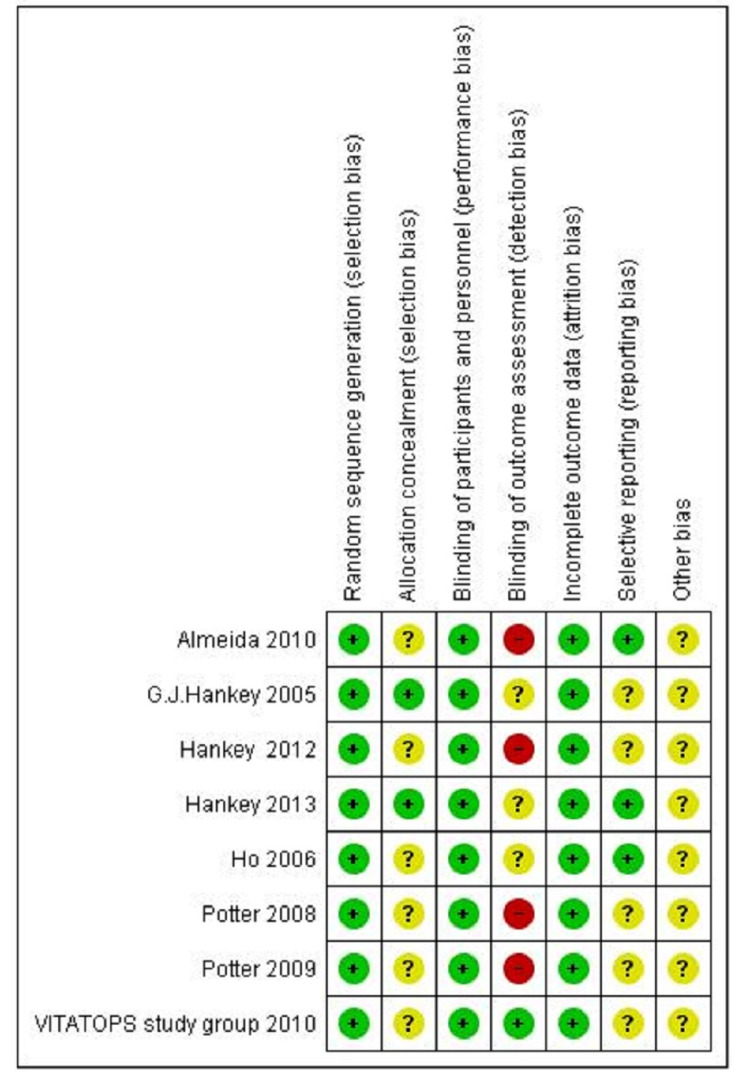
Risk of bias summary Risk of bias summary: review author's judgements about each risk of bias item for each included study

The risk-of-bias assessment was done by the authors NK and PY independently. The Cochrane risk-of-bias assessment tool guidelines by Higgins et al. [12] were used. The risk of bias graph and the risk of bias summary for each selected RCT include selection, performance, detection, attrition, reporting, and other biases (Figures 2-3).

All studies were denoted in the form of low, high, and unclear risk for each component. Any difference in opinion between the two independent authors was resolved by consensus. Heterogeneity was assessed by I^2^ statistics [13] of treatment effects among selected trials assessed with the Chi-square test. 

The complete data derived from the included trials is tabulated in Table 2. 

**Table 2:** Characteristics of the included trials NA: not available

S. N	Author Year	Exp. group / Control group	Exp. Age/ Control Age Mean (SD)	Exp. Male/ Control Male	Treatment group	Control group	Settings
1	Hankey, 2005 [14]	143/ 142	65.4 (13.8) / 65.1 (12.2)	63% / 67%	VITATOPS (Folic acid-2mg, B6=25 mg, B12=0.5mg)	Placebo	India, New Zealand, UK
2	Ho, 2006 [15]	169/ 167	62.1 (11.4) / 60.9 (11.3)	67.3% / 62.7%	VITATOPS (Folic acid-2mg, B6=25 mg, B12=0.5mg)	Placebo	India, New Zealand, UK
3	Potter, 2008 [16]	83/79	65 (11) / 64 (14)	73% / 56%	VITATOPS (Folic acid-2mg, B6=25 mg, B12=0.5mg)	Placebo	India, New Zealand, UK
4	Potter, 2009 [17]	15/13	73 (7) / 67 (10)	73 % / 77%	VITATOPS (Folic acid-2mg, B6=25 mg, B12=0.5mg)	Placebo	India, New Zealand, UK
5	Almeida, 2010 [18]	90/86	63 (11.4) / 61.2 (15.4)	31.5% / 44.4%	VITATOPS (Folic acid-2mg, B6=25 mg, B12=0.5mg)	Placebo	Western Australia
6	The VITATOPS Trial Study Group, 2010 [19]	4089/4075	62.5 (12.6) / 62.6 (12.4)	64% / 64%	VITATOPS (Folic acid-2mg, B6=25 mg, B12=0.5mg)	Placebo	India, New Zealand, UK
7	Hankey, 2012 [20]	734/729	61 (13.3) / 61.3 (13.0)	60.9% / 65.2%	VITATOPS (Folic acid-2mg, B6=25 mg, B12=0.5mg)	Placebo	India, New Zealand, UK
8	Hankey, 2013 [21]	244/237	NA	NA	VITATOPS (Folic acid-2mg, B6=25 mg, B12=0.5mg)	Placebo	India, New Zealand, UK

The randomized sequence methods were properly described among all included trials. The allocation concealment was properly described between two trials (Hankey 2005 [14] and Hankey 2013 [21]), whereas six trials showed unclear status. A double-blinded design was mentioned in all eight trials. Blinding of outcome assessors was described only in one study (The VITATOPS Trial Study Group 2010 [19]), whereas four trials had a high risk-bias (Almeida 2010 [18], Hankey 2012 [20], Potter 2008 [16], and Potter 2009 [17]), followed by three trials with unclear status (Hankey 2005 [14], Ho 2006 [15], and Hankey 2013 [21]). Data was well described in three trials (Almeida 2010 [18], Ho 2006 [15], and Hankey 2013 [21]), whereas five trials showed unclear selective reporting of study data.

The methodological quality summary of each methodological item as per GRADEpro software has been shown in Table 1.

Data analysis

The final statistical analysis was performed according to the protocol in the latest edition of the Cochrane Handbook for Systematic Reviews of RCT and using the RevMan Manager version 5.4 (The Nordic Cochrane Centre, The Cochrane Collaboration, Copenhagen).

Continuous data of homocysteine outcomes was in the form of mean difference (MD) with 95% CI. Events of stroke, cardiovascular events, and vascular death were analyzed as dichotomous variables represented in the form of risk ratios (RR) with 95% CI used to assess the effect of combined vitamin B6, B9, and B12 supplementations on events of fatal and non-fatal stroke, fatal or non-fatal cardiovascular disorders such as myocardial infarction, coronary artery disease, and vascular death among each group. Heterogeneity was assessed by I^2^ statistics [13] of treatment effects among selected trials assessed with the Chi-square test. The researchers categorized I^2^ of 0% as no heterogeneity, 50% as minimal heterogeneity, and >50% as substantial heterogeneity. The researchers used a fixed-effect model for the final meta-analysis with a significant p-value of 0.05 and an I^2^ statistic ≤50% (with nonoverlapping of CI in forest plot suggestive of heterogeneity). All p-values were two-sided with statistically significant at ≤0.05. The potential publication bias was assessed by plotting the funnel graph for the mean difference with standard error to analyze the homocysteine level's primary endpoint. Sensitivity analysis was also performed to assess each RCT's influence and for excluding the trials with enormous effects that were causing heterogeneity in the final result, one by one. Planned stratified analysis was performed based on age, pathological subtype, and cause of ischemic stroke.

## Results

Selection and characteristics of the studies

Initially, in the search strategy, the 2020 guidelines of PRISMA [11] were used for making the flow diagram that reported, screened, excluded, and finally, included. Ninety-one studies were included using the PICO format (PubMed-43, Embase-28, Clinical Key-20), and 83 studies were excluded (as shown in Figure 1). Hence, eight full-text RCTs qualified after the screening, which met all eligibility criteria of this meta-analysis.

Details of the eight included trials’ characteristics have been shown in Table 2. A total of 8513 participants from the eight trials with pre-existing stroke conditions were the sub-study of VITAmins TO Prevent Stroke trial (VITATOPS). Five trials had the complete data for the primary endpoint of homocysteine, whereas two of them were selected for combined stroke, myocardial infarction, and vascular death; three of them had reported secondary endpoints for recurrence events of stroke; two of them for reporting cardiovascular events; and two trials for vascular death among both groups.

The sensitivity analysis which was done by omitting the study done by Almeida et al. 2010 [18] showed a significant difference in heterogeneity found with a mean difference of -3.58 (95% CI -3.70 to -3.46, p=<0.00001), for heterogeneity (I^2^=95%, p<0.00001) (Table 3). Similarly, removing one more study done by Hankey et al. 2013 [21] also reported significant heterogeneity with a mean difference of -4.48 (95% CI -4.78 to -4.18, p⩽0.00001) for heterogeneity (I^2^=94%, p<0.00001). Hence, these two trials (Almeida et al. 2010 [18], Hankey et al 2013 [21]) were removed and five trials (Hankey et al. 2005 [14], Ho et al. 2006 [15], Potter et al. 2008 [16], Potter et al. 2009 [18], and Hankey et al. 2012 [20]) were included in the pooled analysis.

**Table 3:** Sensitivity analysis for the primary outcome (homocysteine level) RCT: randomized controlled trial; CI: confidence interval; P_s_: p-value for significance; I^2^:study heterogeneity; P_H_:* *p-value for heterogeneity

The omitted RCT	Mean difference (95% CI)	P_s_	I^2^	P_H_
Almeida, 2010 [18]	-3.58 (-3.70, -3.46)	<0.00001	95%	<0.00001
Hankey, 2013 [21]	-4.48 (-4.78, -4.18)	<0.00001	94%	<0.00001
Potter, 2008 [16]	Included	-	-	-
Potter, 2009 [17]				
Ho, 2006 [15]				
Hankey, 2012 [20]				
Hankey, 2005 [14]				
Overall effect	-3.84 (-4.17, -3.51)	<0.00001	0%	0.96

**Figure 4:**
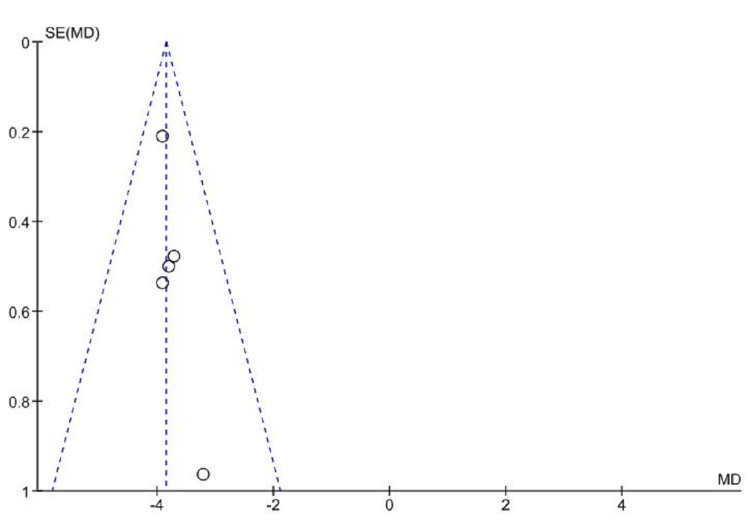
Funnel plot showing the effect of vitamin B supplementations on the homocysteine levels SE(MD): standard error (mean difference)

Funnel plots showed no substantial asymmetry from meta-analysis data to assess the effect of combined Vitamin B6, B9, and B12 supplementations on a mean difference of homocysteine level (Figure 4). The funnel plot of the mean difference of homocysteine showed no significant evidence of publication selection bias.

Primary outcome

Effect of Vitamin B on Homocysteine

**Figure 5:**
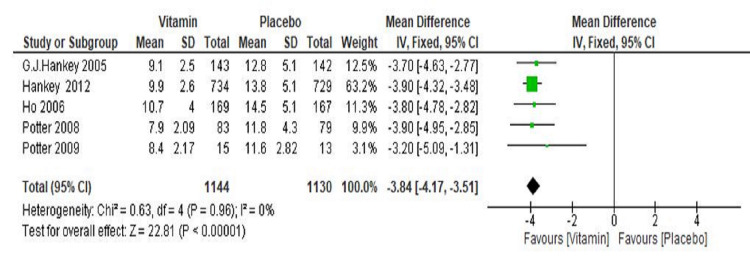
Forest plot showing the effect of vitamin B supplementations on homocysteine level CI: confidence interval; SD: standard deviation

The total pooled result from the five RCTs (Hankey et al. 2005 [14], Ho et al. 2006 [15], Potter et al. 2008 [16], Potter et al. 2009 [17], and Hankey et al. 2012 [20]) showed a significant reduction in the mean homocysteine levels as compared to the placebo group with MD -3.84 (95% CI -4.17 to -3.51; p≤0.00001) (Figure 5). A total of 1144 participants were in the vitamin group, whereas 1130 were in the placebo group. Overall significant heterogeneity was not found in the pooled analysis (I^2^=0%, p=0.96).

Secondary Outcome

Effect of Vitamin on Combined Stroke, Myocardial Infarction, and Vascular Death

**Figure 6:**
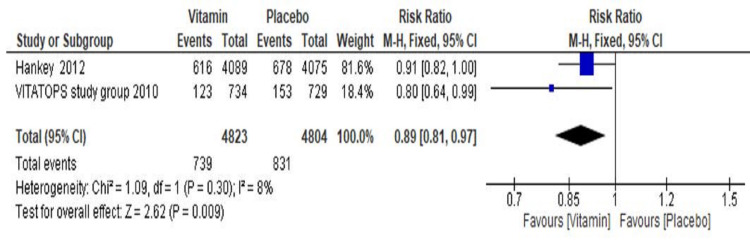
Forest plot showing the effect of vitamin B supplementations on recurrence of combined stroke with myocardial infarction and vascular death events CI: confidence interval

The pooled result of the two included trials (The VITATOPS Trial Study Group 2010 [19] and Hankey et al. 2012 [20]) which received vitamin group showed a significant 11% risk reduction ofcombined stroke, myocardial infarction, and vascular death events as compared to the placebo group (RR=0.89, 95% CI 0.81 to 0.97; p=0.009). A total of 739 events (15%) occurred among 4823 participants in the vitamin group compared to 831 events (17%) among 4804 participants in the placebo group. Overall, significant heterogeneity was not found in this analysis' pooled results (I^2^=8%, p=0.30) (Figure 6). A sensitivity analysis could not be performed as only two trials were included for this secondary endpoint assessment analysis.

A stratified analysis (Table 4) showed no significant difference among subgroups of age <60 years and >60 years for reducing the risk of combined stroke, myocardial infarction, and vascular death events (I^2^=0%, p=0.73). Similarly, no significant difference was found among the pathological subtypes for both ischemic and hemorrhagic stroke for reducing the risk of combined stroke, myocardial infarction, and vascular death (I^2^=28%, p=0.14). Contrary to these findings, a significant difference was found among three subgroups of stroke causes including large artery disease, small artery disease, and embolism from the heart. Small artery disease had a significant risk reduction of 21% among the combined stroke, myocardial infarction, and vascular death events in the vitamin group to placebo (I^2^=0%, p=0.04).

**Table 4:** Stratified analysis of pooled estimates of the risk ratio for combined stroke with myocardial infarction and vascular death *p-value <0.05 considered as significant.

Subgroups	No. of RCTs	No. of participants	Risk ratio (95% CI), p-value	I^2^	p for heterogeneity	
					Within subgroup	Between subgroup
Age						
<60 years	2	4054	0.87 (0.73-1.03), 0.11	0%	0.58	0.73
>60 years	2	5573	0.90 (0.81-1.00), 0.04	0%	0.37	
Pathological subtype of stroke						
Ischemic stroke	2	7166	0.96 (0.86-1.08), 0.50	0%	0.42	0.14
Haemorrhagic stroke	2	2371	0.76 (0.56-1.02), 0.07	28%	0.24	
Cause of ischemic stroke						
Large artery disease	2	3251	1.05 (0.91-1.22), 0.47	3%	0.31	0.04*
Small artery disease	2	2965	0.79 (0.67-0.94), 0.006	0%	0.71	
Embolism from heart	2	611	0.95 (0.71-1.26), 0.73	0%	0.45	

Effect of Vitamin B on Stroke

**Figure 7:**
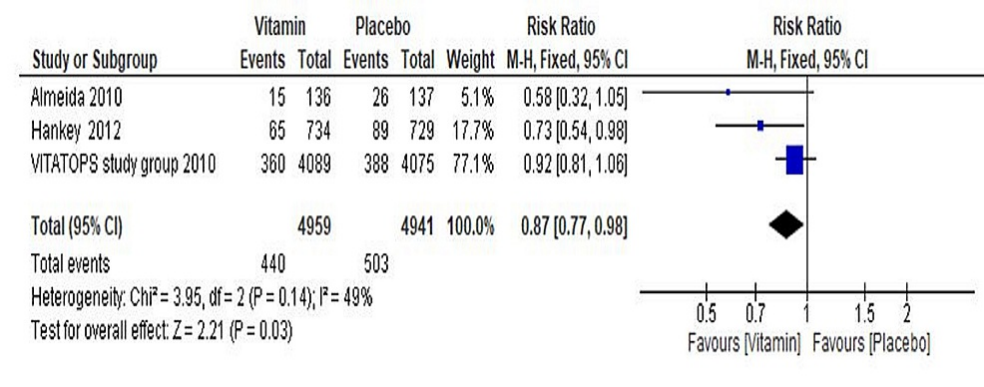
Forest plot showing the effect of vitamin b supplementations on recurrence of stroke events CI: confidence interval

The final result of three trials (Almeida et al. 2010 [18], The VITATOPS Trial Study Group 2010 [19], and Hankey et al. 2012 [20]) showed a significant reduction of 13% in the risk of stroke as compared to the placebo group (RR=0.87, 95% CI 0.77 to 0.98, p=0.03) (Figure 7).

A total of 440 events (8%) among 7959 participants were reported in the vitamin group, whereas 503 events (10%) among 4941 participants were reported in the placebo group. Overall, significant heterogeneity was not found in pooled analysis (I^2^=49%, p=0.14). The sensitivity analysis did not show any substantial change in risk ratio and heterogeneity.

Effect of Vitamin B on Cardiovascular Disorders

**Figure 8:**
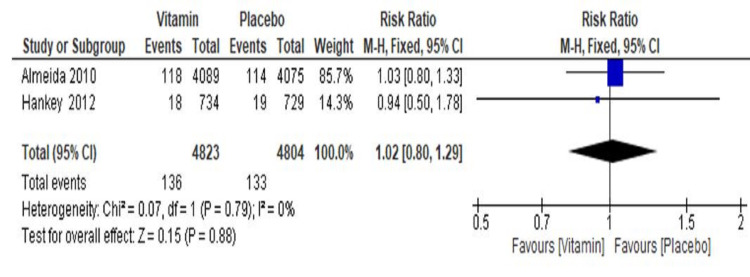
Forest plot showing the effect of vitamin B supplementations on recurrence of cardiovascular events CI: confidence interval

The pooled result from two trials (Almeida et al. 2010 [18] and Hankey et al. 2012 [20]) showed no significant risk reduction for cardiovascular disorder events in the vitamin group as compared to the placebo group (RR=1.02, 95% CI 0.80 to 1.29, p=0.88) (Figure 8).

A total of 136 events (2.8%) among 4823 participants were reported in the vitamin group, whereas 133/4804 events (2.7%) were reported in the placebo group. Overall, significant heterogeneity was not found in pooled results (I^2^=0%, p=0.79). A sensitivity analysis was not possible as only two trials were included in analyzing this secondary outcome for the risk of cardiovascular disorders. 

Effect of Vitamin B on Vascular Death

**Figure 9:**
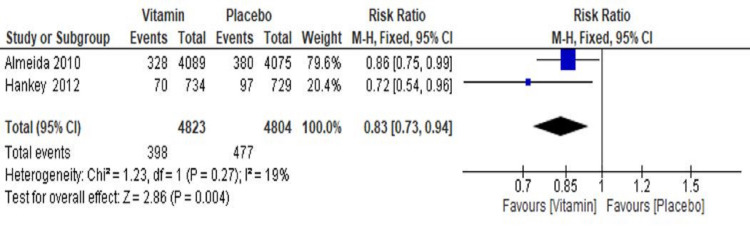
Forest plot showing the effect of vitamin B supplementations on vascular death

The results of two trials (Almeida et al. 2010 [18] and Hankey et al. 2012 [20]) showed a significant reduction of 17% in the risk of vascular death events in the vitamin group as compared with the placebo group (RR=0.83, 95% CI 0.73 to 0.96, p=0.004) (Figure 9).

A total of 8.3% vascular death events (398/4823) were reported in the vitamin group, whereas it was 10% of the events (477/4804) in the placebo group. Overall, significant heterogeneity was not found in the pooled analysis (I^2^=19%, p=0.27). A sensitivity analysis was not possible as only two trials were included in analyzing this secondary outcome for vascular death risk. 

## Discussion

This meta-analysis included eight RCTs with 8513 participants analyzing the effect of vitamin supplementation on homocysteine levels and stroke risk, cardiovascular disorders, and vascular death. This review showed a significant reduction of homocysteine levels among stroke participants. The researchers discussed the effect of vitamin B6, B9, and B12 supplementation under major subheadings of the following variables: homocysteine; combined stroke, myocardial infarction, vascular death; cardiovascular disorders; and vascular death. 

Effect on homocysteine

This meta-analysis indicated a significant reduction of homocysteine level by a mean difference of -3.84 among the vitamin group compared to the placebo group. Similarly, a meta-analysis conducted in 2012 [22], with a total of five trials with 4967 participants with cardiovascular disorders and kidney disease, showed a mean difference in homocysteine levels between folic acid-treated and control participants was -4.97. This study's findings were also supported by trials done in 2013 [21] and the HOPE-2 Trial conducted in 2009 [23], 2006 [15], 2005 [14] for significantly reducing homocysteine levels. Thus, this meta-analysis proved that vitamins play a vital role in the reduction of homocysteine levels. The reduction in homocysteine helps stroke patients in reducing the length of hospitalization and enhances earlier recovery.

Combined effects on stroke, myocardial infarction, and vascular death

The study result revealed a significant reduction of 11% in the risk of combined events of stroke, MI, and vascular death among the vitamin group compared to the placebo group. In contrast, a previous meta-analysis conducted in 2012 [22], with 10 trials, found that the folic acid supplementations group reported 363 cardiovascular events among 1427 participants. This study's findings showed no reduction in combined events of stroke, myocardial infarction, and stroke on sub-group analysis in the folic acid group compared to the comparator group among patients with cardiovascular disorders and kidney diseases (RR=0.99, p=0.45). Inconsistent with the above findings, a sub-group analysis in 2010 [19] supported the prevention of recurrence rates of stroke, cardiovascular disorders, and vascular death. Thus, this meta-analysis proved that vitamins positively reduced 11% of major cardiovascular disorders in a combined form. The reduction in risk of recurrence of major cardiovascular disorders helps stroke patients to prevent future rehospitalization and family burden related to a disease condition.

Effect on stroke

The study's results suggested that vitamin B reduced the risk of stroke by 13% compared to the placebo group. Similarly, a previous meta-analysis in 2016 [24] with 20 trials reported 3164 events among 77816 participants a significant reduction of 10% in the risk of stroke in the folic acid group as compared to the comparator group among patients with CVD, CHD, or stroke (p=0.002; RR=0.90). Similarly, a meta-analysis in 2017) [25] that included 11 RCTs with 2826 stroke events in the total population of 65790 showed a 10% reduction in the risk of stroke among patients with CVDs (p=0.005; RR=0.90). In line with this study's findings, the previous Cochrane meta-analysis in 2017 [26] included 10 trials with 2021 Stroke events from 44224 CVD participants showed a significant reduction of 10% in the risk of stroke. Similarly, in 2010 [27], a meta-analysis that included 13 RCTs, with 784 events among 20415 participants in the folic acid group and 791 events among 18590 participants in the control group, showed no statistically significant difference in preventing the risk of stroke (p=0.16; RR=0.92).

Contrary to the above findings, a meta-analysis in 2012 [22] of eight trials reporting 282 events of stroke among 8586 participants showed no significant reduction in the risk of stroke among the folic acid group as compared to the comparator among cardiovascular events with kidney disease participants (p=0.821; RR=0.97). Contrary to this study's findings, a meta-analysis conducted in 2011 [28] that included 12 RCTs with 2001 events of stroke from 38015 participants showed no evidence of risk reduction among the folic acid group compared to the placebo group (p=0.073; RR=0.89).

The study's results prove that vitamins play an essential role in reducing stroke risk by 13%. Most of the previous studies supported this study's findings but a few had contradictory findings. The reduction in risk of recurrence of the stroke helps stroke patients reduce recovery days after discharge from the hospital. Vitamin supplementation has proven to have a beneficial effect on preventing secondary small artery stroke among these patients, ultimately reducing cost and family burden due to rehospitalization. However, stroke patients had many comorbidities, which led patients to recurrence of stroke if good nutritional status and other factors were not addressed. Vitamin therapy helps stroke patients with altered homocysteine levels in effectively preventing future events of recurrent stroke.

Effect on cardiovascular disorders

The finding showed no significant risk reduction of cardiovascular disorders among the vitamin group than the placebo group, consistent with a previous meta-analysis [22]. The findings of 10 trials reporting 3045 cardiovascular events among 10 863 participants showed that homocysteine-lowering therapy had no overall effect on cardiovascular events among patients with cardiovascular disorders and kidney disorders (p=0.902; RR=0.96). Similarly, Cochrane's meta-analysis [26], which included 12 trials with 3078 MI events from 44199 CVD participants, showed no significant effect of homocysteine-lowering therapy on MI (p=0.56; RR=1.02) among the homocysteine-lowering therapy group. In support of this study's findings, a meta-analysis conducted in 2011 [28] that included 12 RCTs with 8238 events of cardiovascular events from 38015 participants showed no evidence of risk reduction in CVD events, (p=0.096; RR=0.98).

Contrary to these findings, a meta-analysis in 2016 [24] of 22 RCTs with 74346 participants and 9739 CVD events showed a significant reduction of 4% in the risk of cardiovascular events in the folic acid group as compared to comparator among patients of CVD, CHD, or stroke (p=0.02, RR=0.96). In continuation with these findings, a meta-analysis done in 2009 [23] including two trials with 306 major cardiovascular events from 2914 participants showed a significant increase in risk in trial for CVD events in the folic acid plus vitamin B12 group as compared to the placebo group after adjusting confounding variables (p=0.004; HR (hazard ratio)=1.31).

This meta-analysis did not prove that vitamins had a key role in reducing the risk of cardiovascular events among stroke patients. Two trials supported our study findings related to preventing cardiovascular disorders, whereas two trials revealed contradictory findings related to its beneficial effects. Hence, a few more trials are required to assess vitamin B therapy's effect on the risk of recurrence of cardiovascular disorders among stroke patients. In this meta-analysis, the risk of cardiovascular events was not significantly prevented among stroke patients, which is contradictory to the findings in other variables such as homocysteine, stroke, and death.

Effect on vascular death

This meta-analysis showed a 17% reduction in vascular death among the vitamin groups compared to the placebo group, which is contradicted by a previous meta-analysis [22]. This includes six trials that reported 473 vascular death events among 5968 participants that showed no significant reduction in vascular death events in the folic acid group compared to the comparator among patients with cardiovascular disorders and kidney diseases (p=0.865; RR=0.97). Contrary to this study's findings, a previous meta-analysis [29] that included two trials with 226 cardiovascular death events from 3011 participants showed no significant long-term increase in risk after adjusting confounding variables (p=0.11; HR=1.20).

Thus, this meta-analysis proves that vitamins play a crucial role in reducing vascular death events by 17%. The reduction in risk of recurrence of vascular death helps stroke patients survive longer with their loved ones. Cost-effective treatment with vitamin therapy helps to alleviate illness and increase the lifespan of stroke patients.

Strengths and limitations

This meta-analysis includes only RCTs that exclude the majority of confounding variables that can affect study results. This meta-analysis showed an effect of vitamin B supplementation exclusively on stroke patients for assessing change in homocysteine levels and major cardiovascular events. Neither overall heterogeneity nor publication bias was found among selected trials of this meta-analysis for generating the current evidence.

The researchers could not find treatment compliance and dose-dependent relationship due to the unavailability of sufficient data, which may reduce homocysteine levels and risk of recurrence of stroke. The researchers could not match the duration of follow-up between all included trials, which could significantly affect reducing homocysteine levels according to the length of the provided intervention.

## Conclusions

This meta-analysis presented substantial evidence proving the beneficial effect of vitamin B supplementation, especially among stroke patients, in lowering homocysteine with no documented side effects. The evidence proved that vitamin B supplementation effectively reduces homocysteine levels and the risk of stroke and vascular deaths. However, no risk reduction was observed for cardiovascular events among the vitamin group. This treatment is highly recommended in clinical settings, which will become a cost-effective strategy for preventing stroke risk, hence relieving the burden of stroke across the globe by reducing homocysteine levels among stroke patients. The researchers recommend a few more trials with a large sample size and a well-powered study design to observe the effect of homocysteine-lowering therapies on hyperhomocysteinemia at baseline and the risk of cardiovascular events, especially among stroke patients.

## Appendices

PubMed search strategies-43

Filters: Abstract, Clinical Trial, Humans, English, Female, Male, MEDLINE, Adult: 19+ years

("stroke"[MeSH Terms] OR "stroke"[All Fields]) OR "strokes"[All Fields]) OR "stroke s"[All Fields]) OR "stroke"[MeSH Terms]) AND ("vitamin b complex"[Pharmacological Action] OR "vitamin b complex"[MeSH Terms]) OR "vitamin b complex"[All Fields]) OR "vitamin b"[All Fields]) AND ("supplemental"[All Fields] OR "supplementating"[All Fields]) OR "supplementation"[All Fields]) OR "supplementation s"[All Fields]) OR "supplementations"[All Fields]) OR "supplemention"[All Fields]) OR "vitamin b 6"[MeSH Terms]) OR "vitamin b 12"[MeSH Terms]) OR "folic acid"[MeSH Terms])) AND ("recurrance"[All Fields] OR "recurrence"[MeSH Terms]) OR "recurrence"[All Fields]) OR "recurrences"[All Fields]) OR "recurrencies"[All Fields]) OR "recurrency"[All Fields]) OR "recurrent"[All Fields]) OR "recurrently"[All Fields]) OR "recurrents"[All Fields]) AND ("stroke"[MeSH Terms] OR "stroke"[All Fields]) OR "strokes"[All Fields]) OR "stroke s"[All Fields]) OR ("recurrance"[All Fields] OR "recurrence"[MeSH Terms]) OR "recurrence"[All Fields]) OR "recurrences"[All Fields]) OR "recurrencies"[All Fields]) OR "recurrency"[All Fields]) OR "recurrent"[All Fields]) OR "recurrently"[All Fields]) OR "recurrents"[All Fields]) AND ("cardiovascular diseases"[MeSH Terms] OR ("cardiovascular"[All Fields] AND "diseases"[All Fields]) OR "cardiovascular diseases"[All Fields]) OR ("cardiovascular"[All Fields] AND "disorders"[All Fields]) OR "cardiovascular disorders"[All Fields]) OR ("myocardial infarction"[MeSH Terms] OR ("myocardial"[All Fields] AND "infarction"[All Fields]) OR "myocardial infarction"[All Fields]) OR ("death"[MeSH Terms] OR "death"[All Fields]) OR "deaths"[All Fields]) OR ("venous thrombosis"[MeSH Terms] OR ("venous"[All Fields] AND "thrombosis"[All Fields]) OR "venous thrombosis"[All Fields]) OR (("deep"[All Fields] AND "vein"[All Fields]) AND "thrombosis"[All Fields]) OR "deep vein thrombosis"[All Fields]) OR ("pulmonary embolism"[MeSH Terms] OR ("pulmonary"[All Fields] AND "embolism"[All Fields])) OR "pulmonary embolism"[All Fields]) AND ("death"[MeSH Terms] OR "death"[All Fields]) OR "deaths"[All Fields]) OR ("homocystein"[All Fields] OR "homocysteine"[MeSH Terms]) OR "homocysteine"[All Fields]) OR "homocysteine s"[All Fields]) OR "homocysteines"[All Fields]) OR "hyperhomocystenemia"[All Fields]) AND ("placeboes"[All Fields] OR "placebos"[MeSH Terms]) OR "placebos"[All Fields]) OR "placebo"[All Fields]) AND ("routine"[All Fields] OR "routinely"[All Fields]) OR "routines"[All Fields]) OR "routinization"[All Fields]) OR "routinize"[All Fields]) OR "routinized"[All Fields]) OR "routinizing"[All Fields]).

EMBASE search strategies-28

‘Stroke’ AND ('cyanocobalamin'/dd OR 'folic acid'/dd OR 'homocysteine'/dd OR 'placebo'/dd OR 'pyridoxine'/dd OR 'vitamin b group'/dd) AND  (2001:py OR 2002:py OR 2003:py OR 2005:py OR  2006:py OR 2007:py OR 2008:py OR 2009:py OR 2010:py OR 2011:py OR 2012:py OR 2013:py OR 2014:py OR 2015:py OR 2016:py OR 2017:py OR 2018:py OR 2019:py) AND ('acute coronarysyndrome'/dm OR 'angina pectoris'/dm OR 'brain ischemia'/dm OR 'cardiovascular disease'/dm OR     'cerebrovascular accident'/dm OR 'heart infarction'/dm OR 'hyperhomocysteinemia'/dm OR 'ischemic heart disease'/dm OR 'recurrent      disease'/dm OR 'transient ischemic attack'/dm OR 'unstable angina pectoris'/dm) AND ‘vascular death’/dm.

Clinical Key search strategies-20

Filters: full text only, randomized controlled trials, 1 January 2010-January 2020

Stroke AND Vitamin B supplementation AND placebo AND homocysteine

Stroke AND Vitamin B supplementation AND placebo AND recurrence of stroke

Stroke AND Vitamin B supplementation AND placebo AND recurrence of cardiovascular disorders

Stroke AND Vitamin B supplementation AND placebo AND death
